# Near-Infrared Heptamethine Cyanine Based Iron Oxide Nanoparticles for Tumor Targeted Multimodal Imaging and Photothermal Therapy

**DOI:** 10.1038/s41598-017-01108-5

**Published:** 2017-05-18

**Authors:** Sejy Lee, Reju George Thomas, Myeong Ju Moon, Hyeong Ju Park, In-Kyu Park, Byeong-Il Lee, Yong Yeon Jeong

**Affiliations:** 1 Department of Radiology, Chonnam National University Hwasun Hospital, Hwasun, 58128 South Korea; 2 Medical Photonics Research Center, Korea Photonics Technology Institute, Gwangju, 61007 South Korea; 3 Department of Biomedical Sciences, Chonnam National University Medical School, Gwangju, 61469 South Korea

## Abstract

Near-infrared fluorescent (NIRF) imaging modality holds great promise for tumor detection and offers several advantages of bioimaging, such as high tissue penetration with less background scattering. The disadvantage of NIRF bioimaging is that it has very low spatial resolution. Thus, the combination of NIRF with magnetic resonance imaging (MRI) is a good option because MRI can provide anatomical information with a higher resolution. Heptamethine cyanine dye (MHI-148) has been reported to have tumor-targeting capability which was used here as the NIRF agent. DSPE-SPION nanoparticles were synthesized by the solvent hydration method and conjugated with MHI-148 dye to form a MRI/NIRF dual imaging probe. The size and charge of the MHI-DSPE-SPION were found to be about 84 ± 6 nm and 3.7 mV by DLS & Zeta Potential analysis. *In vivo* MRI of the SCC7 tumor showed an enhanced accumulation of MHI-DSPE-SPION, peaking at day 1, compared to 4 hrs with the control DSPE-SPION. An *in vivo* photothermal tumor reduction study was done on the SCC7 tumor of BALB/c nude mice. Tumor reduction study showed complete tumor removal after 8 days. In conclusion, MHI-DSPE-SPION can be used as a cancer theranostics material because it provides MRI-optical imaging capabilities and the photothermal therapy (PTT) effect.

## Introduction

Near-infrared fluorescent (NIRF) imaging pertains to optical fluorescent imaging in the NIR wavelength range of 700–1,000 nm, which provides excellent tissue penetration and image sensitivity. However, NIR dyes still have certain drawbacks, including low hydrophilicity, quantum yield, and low detection sensitivity, which make them less appealing for cancer theranostic applications^1, 2^. Recently, general interest has developed in the heptamethine class of NIRF dyes, such as IR-780, IR-783, and IR-808, which exhibit preferential accumulation in addition to a significantly higher signal-to-noise ratio (SNR) in tumors^3–5^. Several studies in this field have been carried out to utilize the special properties of these heptamethine dyes for cancer diagnosis and therapy^4, 6, 7^.

The heptamethine cyanine dye MHI-148 is an analogue of IR-783, which was initially synthesized as an optical imaging agent to detect human kidney cancer^8, 9^. MHI-148 dye-conjugated porous Gd silicate nanoparticles were developed as a NIRF-MRI dual modal contrast agent for LLC/LL2 tumors^10^. In another work, a MHI-148-based PET imaging probe labelled with Cu^64^ was developed as a tumor-targeting agent^11^. These studies showed that the MHI-148 dye has active tumor-targeting properties and enhanced accumulation in tumors compared to control groups.

Magnetic resonance imaging (MRI) is an anatomical imaging technique that gives excellent spatial resolution in images^12^. Superparamagnetic iron oxide nanoparticles (SPION) is a T2-weighted MRI contrast agent and can be effectively loaded or encapsulated by a 1,2-distearoyl-sn-glycero-3-phosphoethanolamine (DSPE)-polyethylene glycol (PEG) lipid-polymer system, thereby increasing the T2 contrast efficiency and bio-stability^13–15^. DSPE-PEG is a biocompatible system that has been used to develop delivery systems for hydrophobic anti-cancer drugs^16, 17^. It has also been used in photothermal applications with indocyanine green (ICG) dye^18^. Amine-functionalized DSPE-PEG gives the nanoparticles the ability to conjugate with carboxyl-terminated small molecules, thereby improving the system by imparting the active targeting properties^19^.

**Figure 1:**
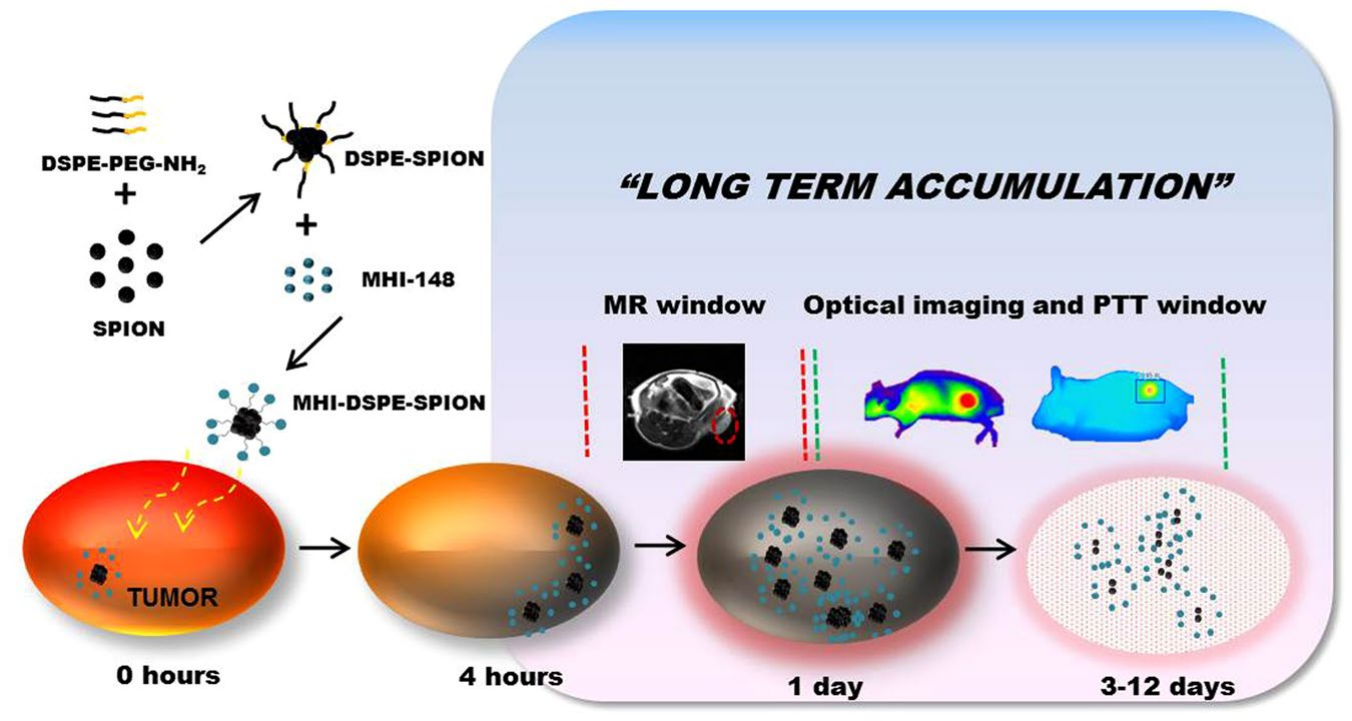
Schematic illustration showing the overall concept of the present study. MHI-DSPE-SPION designed for MRI application by using the magnetism of the nanoparticle core of SPION (black) in addition to optical imaging along with PTT by MHI-148 ligands (green) of the nanoparticle shell (DSPE-PEG).

NIRF imaging produces highly sensitive contrast and very low tissue SNR compared to MRI^12^. NIRF heptamethine dyes, such as IR-780 and IR-820, have shown excellent photothermal effects *in vitro* and *in vivo* with or without nanoparticle conjugation^20^. MHI-148 shows photothermal activity under selective heating due to the absorption of laser energy in addition to high, NIR-induced photothermal conversion efficiency^21^. However, to our knowledge, the photothermal properties of MHI-148 have not been analyzed. The concept of our study is shown in Fig. 1.

The objective of our work was to study the action of MHI-DSPE-SPION as a NIRF-MRI dual mode contrast agent that also shows long-term accumulation and an active targeting ability for MHI-148. This study also aimed to prove the PTT effect of MHI-DSPE-SPION.

## Results and Discussion

**Figure 2:**
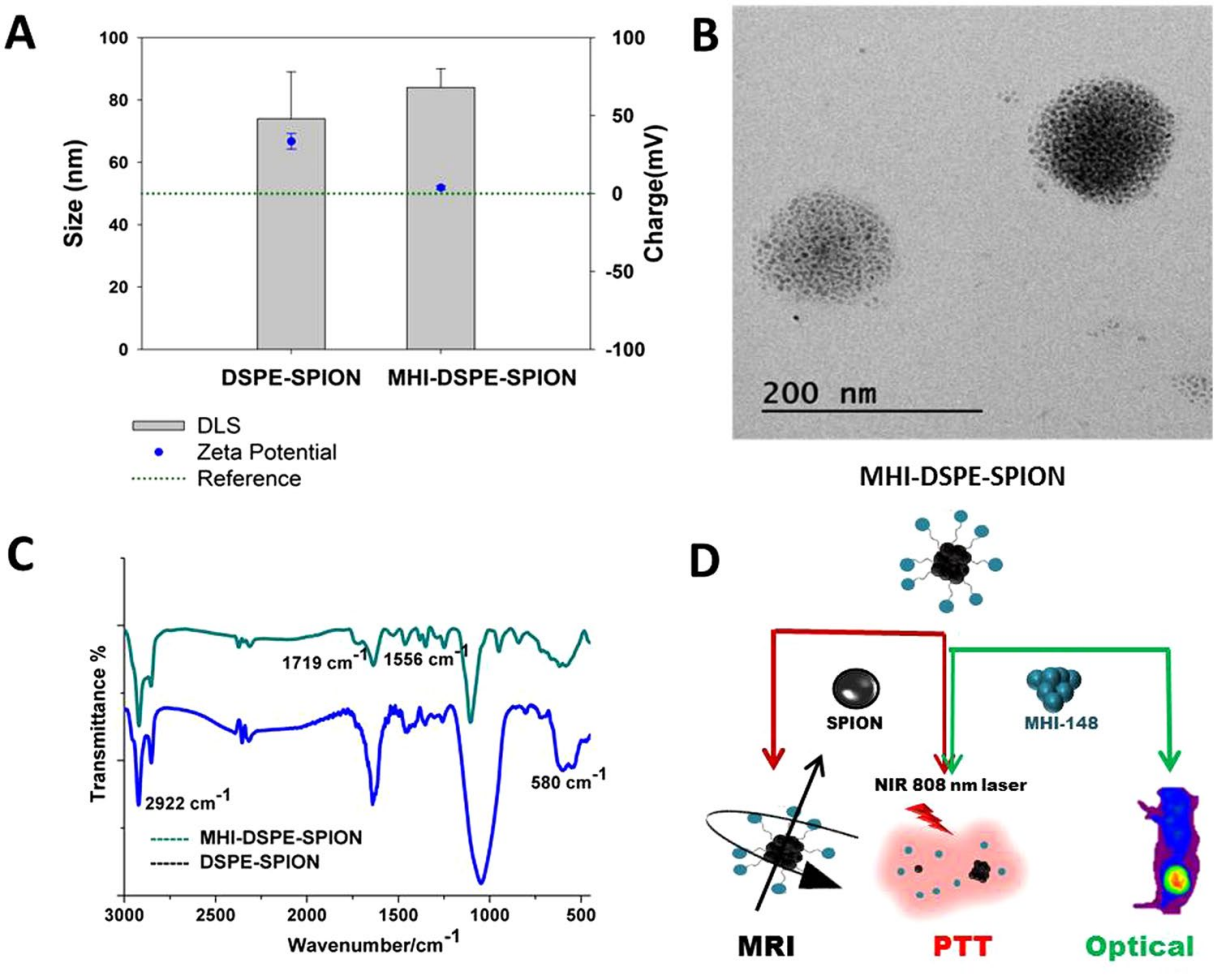
Physicochemical properties of MHI-DSPE-SPION and DSPE-SPION. (**A**) Surface charge and hydrodynamic size, as measured by zeta potential and DLS analysis, respectively. (**B**) MHI-DSPE-SPION morphology, as observed by TEM (**C**) FT-IR was done in the spectral range between 800 and 3000 cm^−1^ for DSPE-SPION and MHI-DSPE-SPION to analyze the bonding between DSPE-SPION and the MHI-148 dye. (**D**) Illustration describing the role of SPION and MHI-148.

Oleic-acid-coated SPION were synthesized via the thermal decomposition method to obtain uniform, 5 nm particles in size. Hydrophobic SPION was loaded onto DSPE-PEG-NH_2_ using the well-established solvent hydration method^15^. The size and zeta potential of DSPE-PEG-SPION were measured to be 74 ± 15 nm and 33.5 mV for 10:4 (DSPE-PEG:SPION weight ratio). Transmission electron microscopy (TEM) was utilized to investigate the morphology of DSPE-PEG-SPION. A spherical morphology was observed by TEM (Fig. 2A&B) with SPION cluster formation which is ideal for MRI contrast enhancement compared to single SPION nanoparticle^22^. MHI-148 was conjugated to DSPE-PEG-SPION by COOH- (MHI-148) and –NH2 (NH2-PEG-DSPE-SPION) bond conjugation with help of EDC/NHS carbodiimide chemistry. The size and zeta potential of MHI-DSPE-SPION were 84 ± 6 nm and 3.7 mV, respectively.

Size is an important factor in tumor accumulation due to its enhanced permeability and retention (EPR) effects in addition to design characteristics such as charge and shape^23, 24^. Recently, ICG-loaded lipid-polymer nanoparticles were prepared by using poly (lactic-co-glycolic acid) (PLGA)-lecithin-PEG to analyze the influence of particle size on effective tumor accumulation and the PTT effect^25^. This report showed that 68 nm nanoparticles showed significant tumor accumulation due to the EPR effect compared to 118 nm particles. They also showed improved tumor retention compared to the smaller 38 nm particles. In our study, the MHI-DSPE-SPION particle size was controlled within the size range of 50–100 nm to obtain a better tumor accumulation for MRI and photothermal therapy.

Charge variation is attributed to effective amine conjugation by MHI-148, as verified by FTIR (Fig. 2C). The FTIR spectrum of MHI-148 has a characteristic band originating at 1550 cm^−1^, from benzene ring vibrations, and at 1719 cm^−1^, originating from C = O stretching of MHI-148. The FTIR spectrum obtained after conjugating DSPE-PEG-SPION with MHI-148 reveals bands at 1719 cm^−1^ and 1556 cm^−1^ ^10^. Additionally PEG_2000_ in MHI-DSPE-PEG and DSPE-PEG showed characteristic peak at 2922 cm^−1^ caused due to CH alkyl stretching^26^ and finally peak at 580 cm^−1^ is due to Fe-O group vibration from SPION^27^. The role of SPION and MHI-148 is clearly shown in (Fig. 2D).

**Figure 3:**
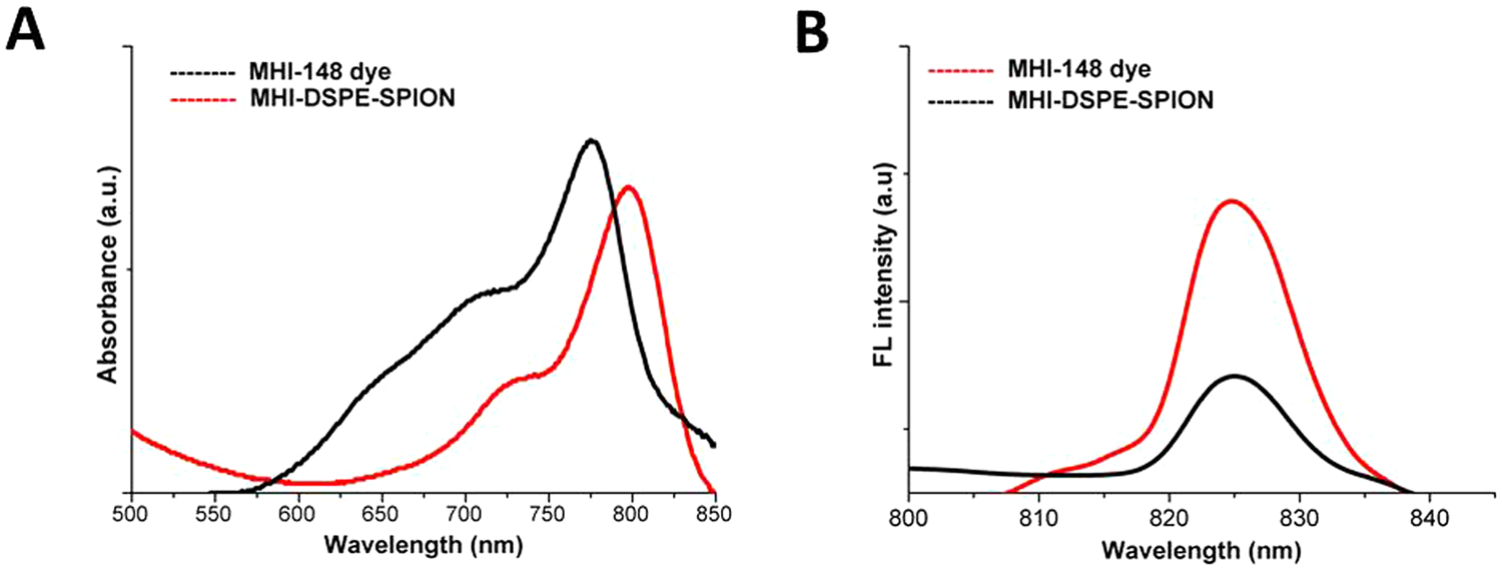
Ultraviolet–visible absorbance and fluorescence of free (**A**) MHI-148 dye and (**B**) MHI-DSPE-SPION nanoparticles.

The UV absorption profile of MHI-DSPE-SPION shows a peak shift from 774 nm to 794 nm, whereas the fluorescence profile remains unchanged (Fig. 3).

**Figure 4:**
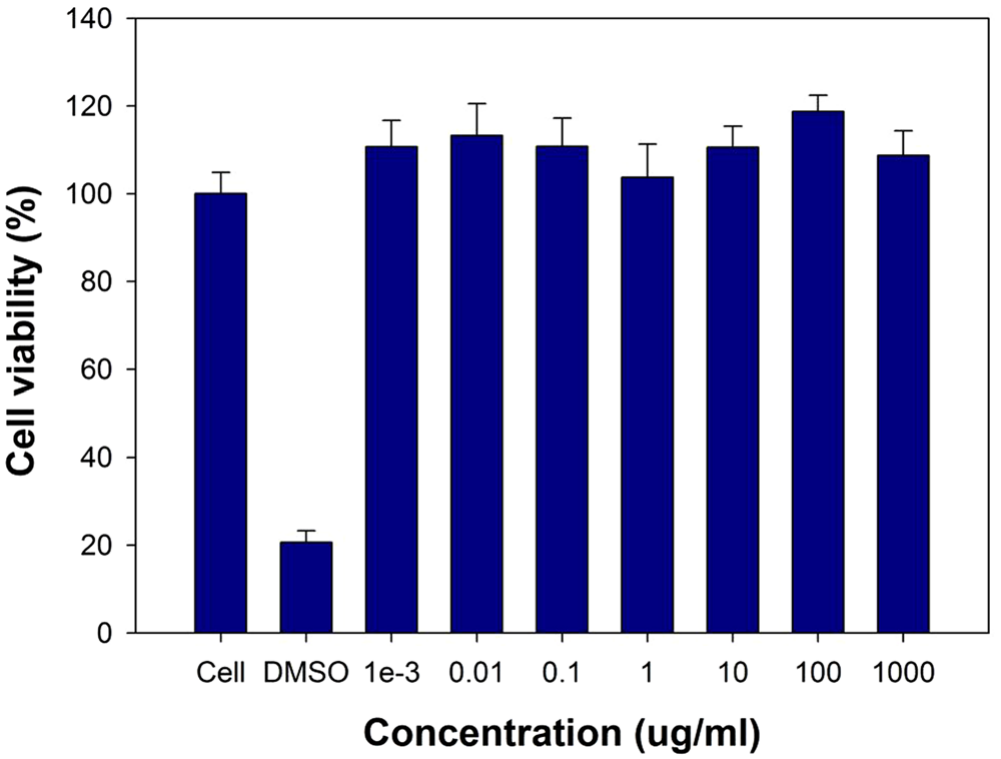
Cytotoxicity of MHI-DSPE-SPION analysed using NIH3T3 cells, as quantified by MTS assay. Mean cell viability of NIH3T3 was used for MTS assay in quadruplicate samples ± SD.

NIH3T3 (murine fibroblast cell line) as a control is used commonly to evaluate cell toxicity. The NIH3T3 cell line was treated with MHI-DSPE-SPION to check for cytotoxic effects. Cells without any treatment was taken as control and DMSO (5 µL per well) added cells as negative control. Compared to DMSO treated cells, which showed only 20% cell viability, MHI-DSPE-SPION treated cells showed no toxicity in NIH3T3 cells even at a high concentration value of 1,000 µg/mL (Fig. 4).

**Figure 5:**
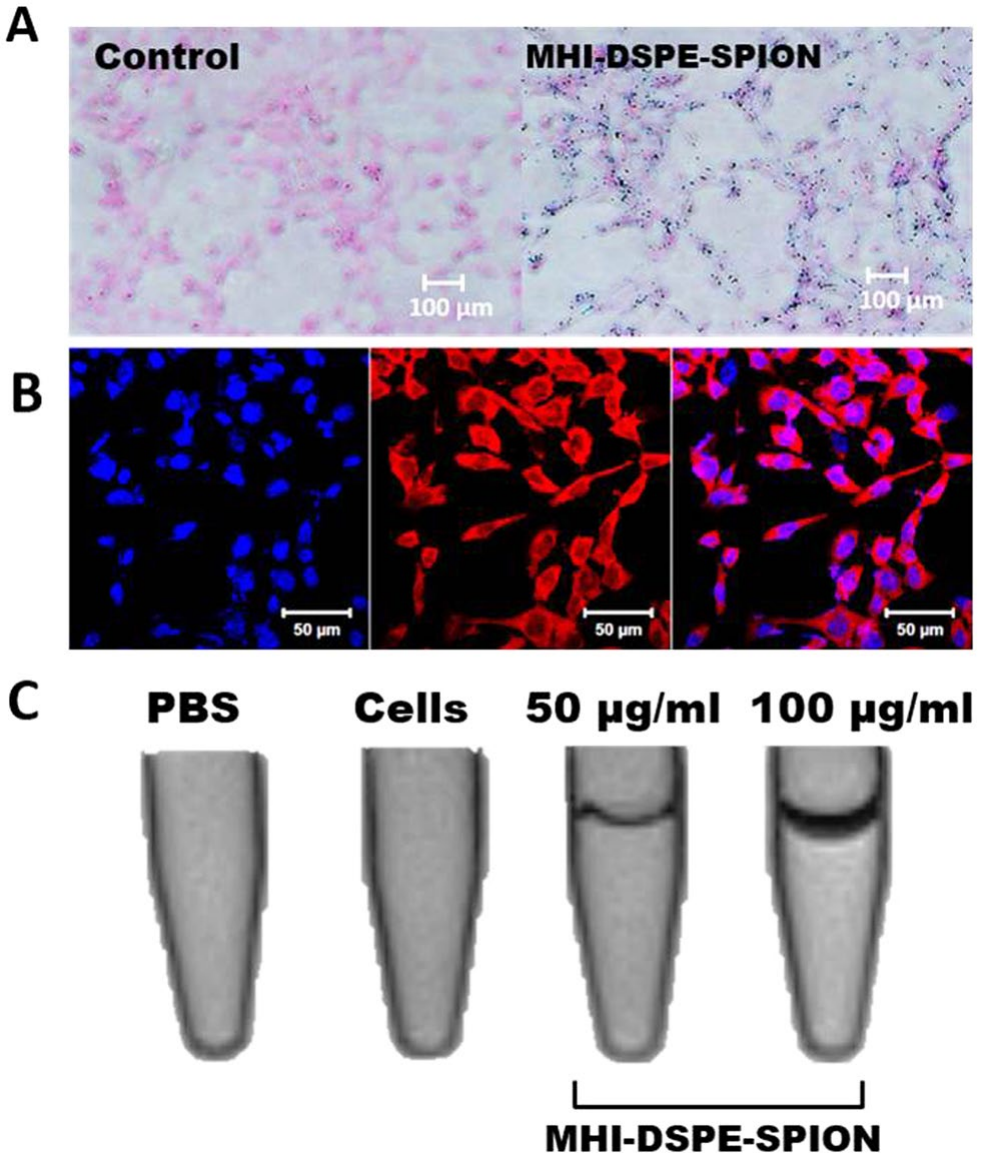
Uptake of MHI-DSPE-SPION in SCC7 cells. The dosage was 50 µg/mL, and the incubation time was 2 hrs. (**A**) Prussian blue staining images show significant uptake of MHI-DSPE-SPION in SCC7 cells compared to the control. (**B**) CLSM image showing MHI-DSPE-SPION (red) and nuclei stained for 40, 6-diamidino-2-phenylindole (DAPI), and a merged image made from NIR and DAPI images. (**C**) *In vitro* phantom tube MRI of SCC7 cells shows a dark band within the tube with the intracellular uptake of MHI-DSPE-SPION. A thicker band was shown for 100 µg/mL dosage, which proves concentration based uptake efficiency increase in SCC7 cells.

Cell uptake was analyzed by confocal laser scanning microscopy (CLSM) and the Prussian blue staining method (Fig. 5A and B). MHI-148 NIR fluorescence was detected by CLSM upon the uptake of MHI-DSPE-SPION into the cancer cells. After 2 h of incubation, the highest NIR fluorescence signals were observed in SCC7 cells. MHI-148 dye uptake in cancer cells is hypothesized to be mediated through organic anionic transporters (OATP) with high accumulation in mitochondria^28, 29^. Although the uptake of MHI-148 free dye by cancer cells has been studied, only a few works have examined MHI-148 dye-conjugated nanoparticle uptake by cancer cells. Here, SCC7 cancer cells are shown to uptake MHI-DSPE-SPION nanoparticles, and the data show conclusive proof of the nanoparticle conjugation-retained natural uptake mechanism of MHI-148.

Prussian blue staining was done to qualitatively analyze SPION uptake in SCC7 cells, which showed a similar uptake profile that clearly demonstrated nanoparticle uptake. MR sensitivity was investigated by T2-weighted MRI of SCC7 cell lines incubated *in vitro* with MHI-DSPE-SPION, with pure PBS and SCC7 cell lines used as controls. In the T2-weighted images, the control cells and PBS showed no contrast enhancement, whereas the treated cells demonstrated a contrast enhancement effect, showing a drop in signal intensity (SI) (dark signal band in tube) due to the T2 shortening effect. This reflects the uptake of MHI-DSPE-SPION into SCC7 cell lines. Thus, MHI-DSPE-SPION can be exploited as an efficient tool for MRI-based cellular imaging due to its effective intracellular delivery and high contrasting capability (Fig. 5C).

**Figure 6:**
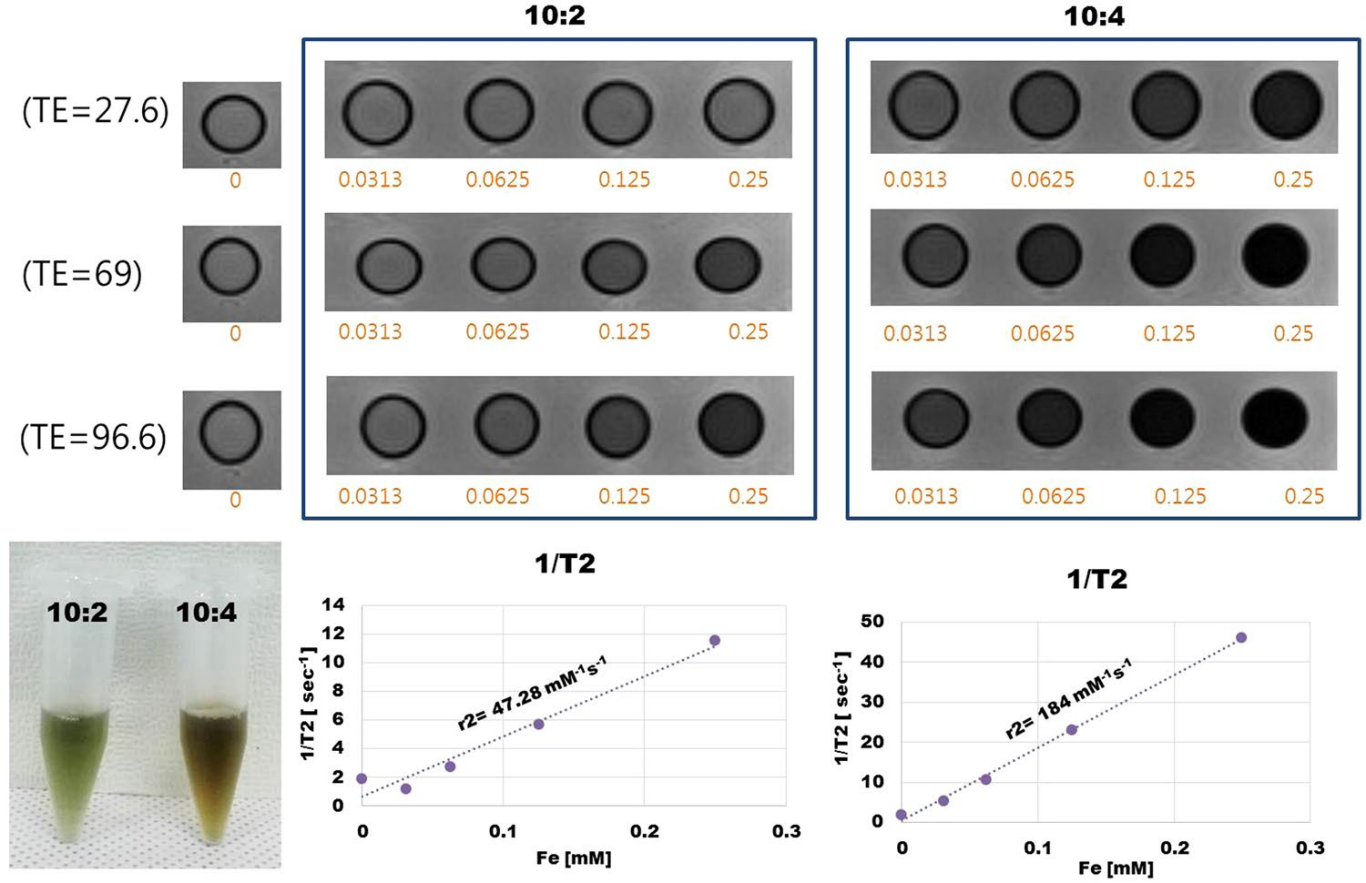
Relaxivity (r2 value) measurements of MHI-DSPE-SPION by linear correlation between the relaxation rate, 1/T2, and the concentration of iron for ratios of 10:2 and 10:4.

Relaxivity measurements of MHI-DSPE-SPION were performed by linear correlation between the 1/T2, relaxation rate, and the concentration of iron. Two loading ratios of DSPE-PEG-NH_2_ to SPION (10:2 and 10:4) were selected for analysis (Fig. 6). The r2 values, calculated from the slopes of the graphs, were found to be 47.28 mM^−1^s^−1^ and 184 mM^−1^s^−1^ for ratios of 10:2 and 10:4, respectively. T2 relaxivity measurements of MHI-DSPE-SPION were performed during MRI analysis. Based on the r2 value obtained for SPION, the 10:4 ratio was selected for further analysis because a higher T2 relaxation contributes to better MR performance (Fig. 6).

**Figure 7:**
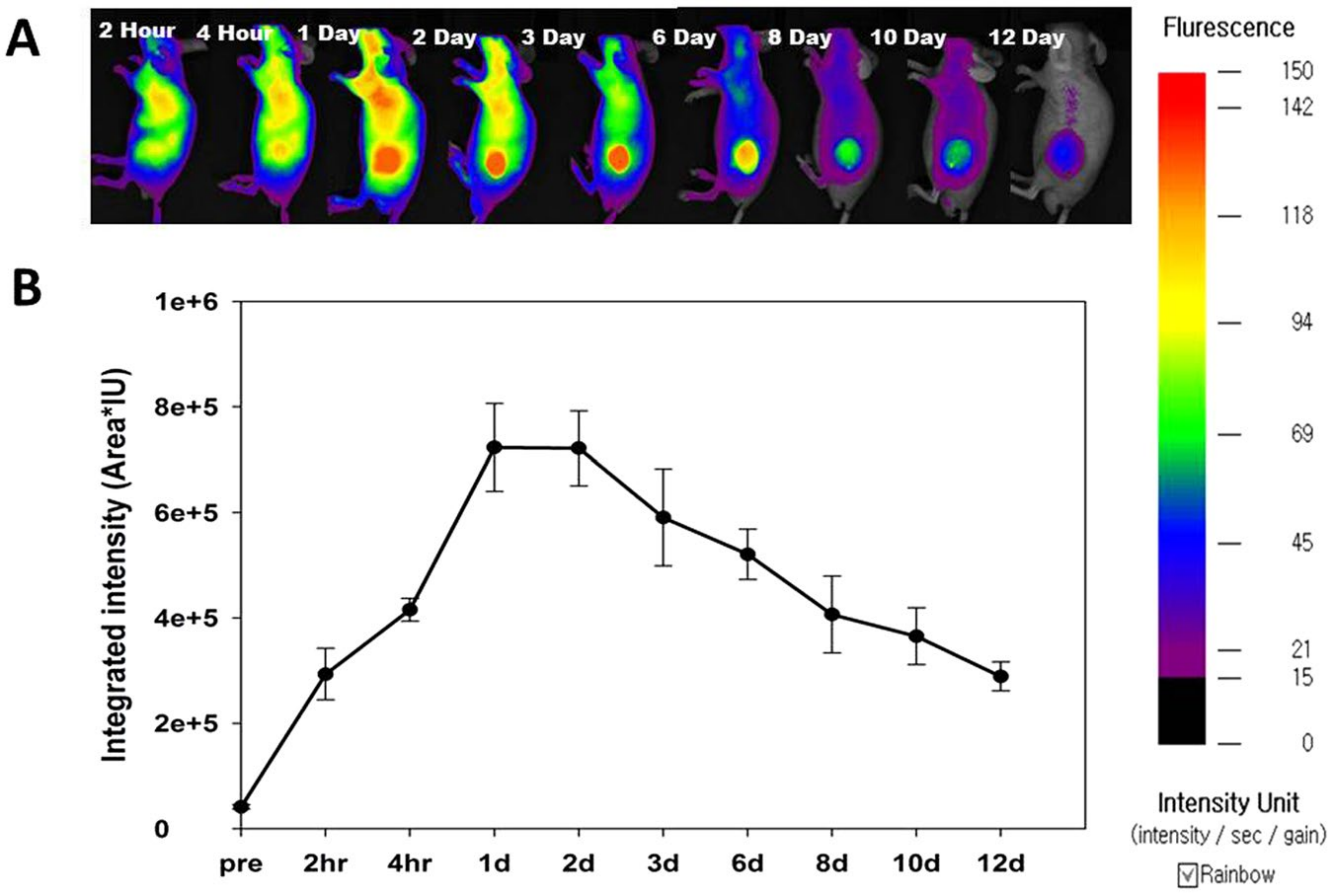
NIR fluorescence and biodistribution for MHI-DSPE-SPION injected BALB/c nude mice *in vivo*. NIR images of mice bearing the SCC7 tumor, captured within 2 hrs after *i.v*. injection of MHI-DSPE-SPION at a dose 10 mg[Fe]/kg. The mice were subjected to fluorescence imaging (NIR) using the FOBI imaging system. (**A**) *In vivo* NIR fluorescence of BALB/c nude mice, measured at different times. (**B**) Quantitative analysis of tumor fluorescence intensity (ROI), measured as integrated intensity (Area*IU).

For *in vivo* biodistribution, MHI-DSPE-SPION was intravenously injected to BALB/c nude mice bearing the SCC7 tumor, at a dose of 10 mg[Fe]/kg. Accumulation in the liver was evident 2 hrs after injection and increased in the tumor at later times. After 1 day, the peak intensity of MHI-DSPE-SPION fluorescence in the tumor was retained until day 2. After 3 days, fluorescence intensity in the tumor diminished but was retained until day 12 (Fig. 7A). The accumulation peak in fluorescence imaging (Fig. 7B) was similar to the SI drop observed for *in vivo* MRI at 1 day.

**Figure 8:**
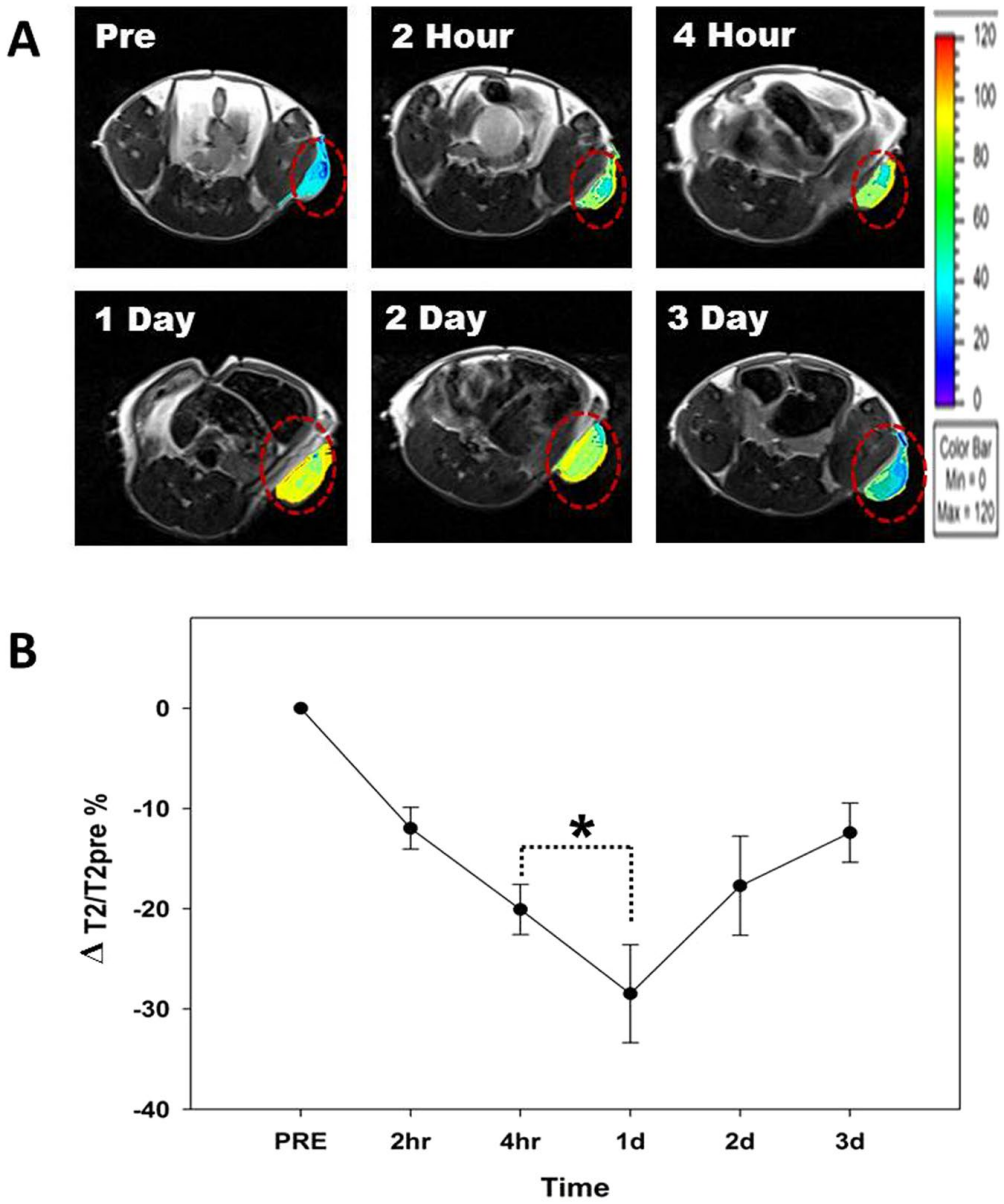
*In vivo* MRI study. (**A**) *In vivo* T2-weighted MRI of MHI-DSPE-SPION. (**B**) Signal change of the SCC7 tumor in T2-weighted MRI, measured at different time points (pre, 2 hrs, 4 hrs, 1d, 2d, 3d) for 10 mg[Fe]/kg. MHI-DSPE-SPION (in PBS) was injected through the tail vein. *P < 0.05 relative to the pre-injection T2 contrast (n = 8).

For the *in vivo* MRI study, MHI-DSPE-SPION and DSPE-SPION were intravenously injected into mice having SCC7 tumor followed by MRI scanning of the mice. The SI of the SCC7 tumor in MRI decreased post-injection of MHI-DSPE-SPION and DSPE-SPION. The contrast enhancement (SI drop) of MHI-DSPE-SPION reached a maximum of 30% at 1 day post-injection, with the SI reaching −10% after 3 days (Fig. 8A and B). The SI of the post-contrast MR image 4 hrs after injection of DSPE-SPION decreased to a maximum of 37% compared with that of the pre-contrast MR image (in Figure S1 of the supplementary information) and reached −10% SI on 1 day. From this result we can infer that MHI-DSPE-SPION have better accumulation property in tumor than DSPE-SPION due to active targeting mechanism. Since active targeting nanoparticle system can accumulate in tumor for more time than passive targeting system^30^. This result coincides with the *in vivo* fluorescence imaging data, in which the SI of the tumor of MHI-DSPE-SPION injected mice group is maximum at 1 day post injection. Iron oxide nanoparticles have tendency of leaching or iron dissolution at lower pH as in tumor environment^31^. We assume that SI drop of MRI at day 2 is probably due to iron dissolution which is not the case for NIR fluorescent agent like MHI-148. Therefore we can see SI drop from day 1 on MRI but not for the *in vivo* fluorescence imaging data where fluorescence signal drop is at much slower rate.

The accumulation of MHI-DSPE-SPION and DSPE-SPION was confirmed by *ex vivo* Prussian blue staining of the SCC7 tumor extracted from mice 1 day after injection at a concentration of 10 mg[Fe]/kg (in Figure S2 of the supplementary information). The amount of iron accumulated in MHI-DSPE-SPION was higher than that of DSPE-SPION in the mouse tumor, validating maximum accumulation at 1 day after intravenous injection of MHI-DSPE-SPION. These results indicate the importance of the MHI-148 dye-conjugated nanoparticles for long-term accumulation. MHI-DSPE-SPION, as reported here, is a potential choice for long-term *in vivo* NIRF and MRI, compared with DSPE-SPION, due to the higher tumor targeting ability of MHI-148.

**Figure 9:**
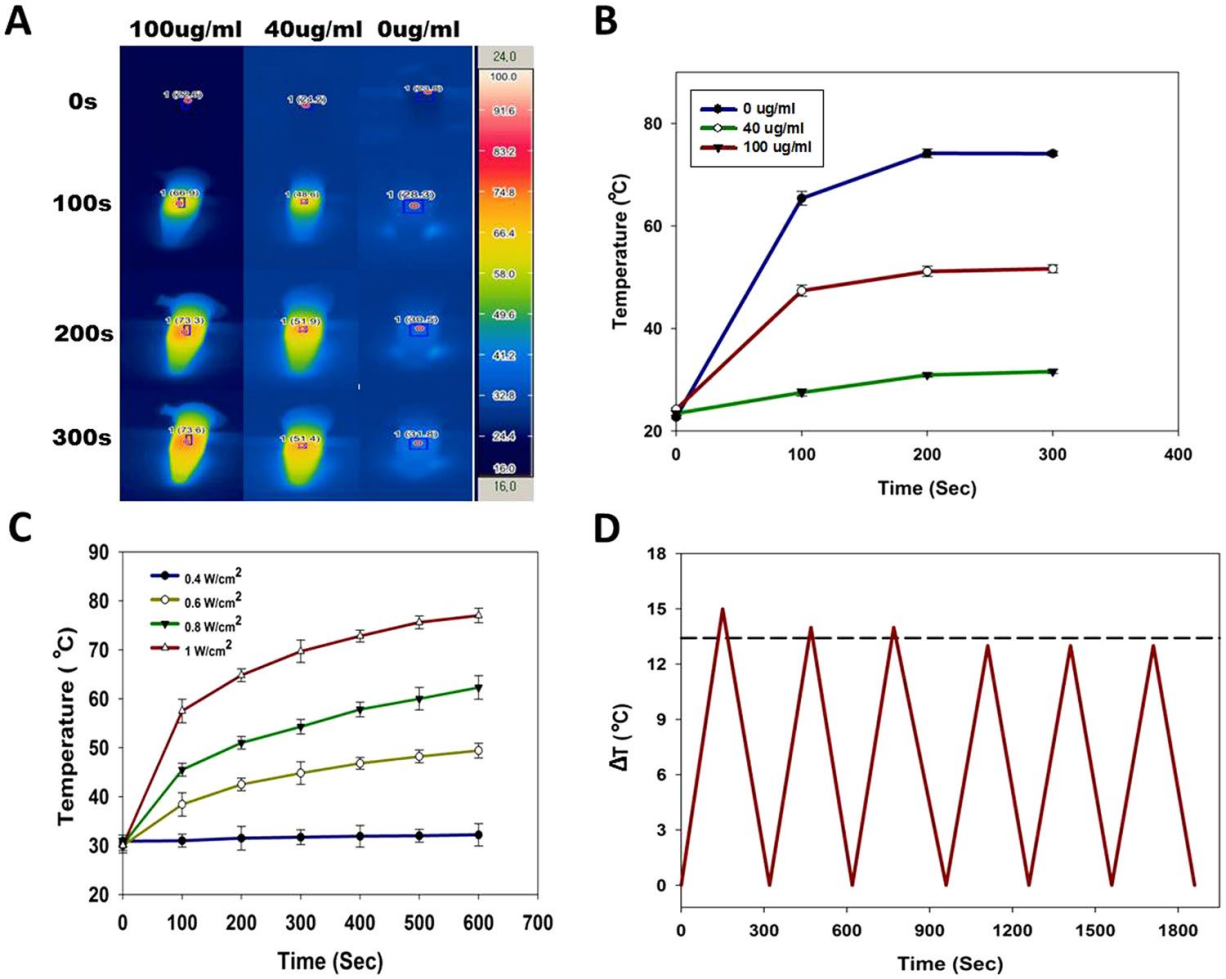
Photothermal studies of MHI-DSPE-SPION. (**A**) Infrared thermo-graphic images of 100 µg/mL, 40 µg/mL and 0 µg/mL MHI-DSPE-SPION after irradiation for 300 sec at 1 W/cm^2^ density. (**B**) Temperature rise after 300 sec of laser irradiation for different concentrations of MHI-DSPE-SPION. (**C**) Temperature rise after 600 sec of laser irradiation for MHI-DSPE-SPION solutions (20 μg/mL) of different laser powers. (**D**) Photothermal stability study of MHI-DSPE-SPION solution.

We studied the NIRF laser light induced heating ability of MHI-DSPE-SPION in PBS by recording the temperature rise. The temperature was monitored with an IR thermal imaging system (Avio IR camera/Thermometer, Shinagawa-ku, Tokyo) (Fig. 9A). Different concentrations of MHI-DSPE-SPION were exposed to an 808 nm laser at a density of 1 W/cm^2^ for 300 sec. An increase in temperature was observed and depended on the concentration of the MHI-DSPE-SPION solution. The temperature of MHI-DSPE-SPION (40 μg/mL [MHI-148]) increased approximately 45 °C within 100 seconds (Fig. 9B). Then, to prove that the NIR laser power also contributed to the increase in temperature, a laser-power-dependent analysis was conducted with 20 μg/mL [MHI-148] MHI-DSPE-SPION (Fig. 9C). The temperature increment of MHI-DSPE-SPION was also found to be dependent on the laser power. The PTT stability of MHI-DSPE-SPION was also observed over five cycles of laser irradiation (Fig. 9D). MHI-DSPE-SPION showed a stable photoheat conversion property.

The temperature variation profile of DSPE-SPION at different concentrations was investigated by continuous laser irradiation. After 2 W/cm^2^ laser irradiation for 15 min, the maximum temperature of 3 mg/mL, 1.5 mg/mL, and 0.5 mg/mL DSPE-SPION in PBS rose to 65 °C, 53 °C, and 36 °C, respectively (in Figure S3 of the supplementary information). This result indicates that SPION can play an important role in photothermal applications based on its concentration. In addition to the photothermal heating effect of MHI-148 in MHI-DSPE-SPION, SPION can support the thermal ablation of the tumor above the threshold temperature of 42 °C via the simultaneous application of DUAL-mode^32^ photothermal and alternating magnetic field (AMF).

**Figure 10:**
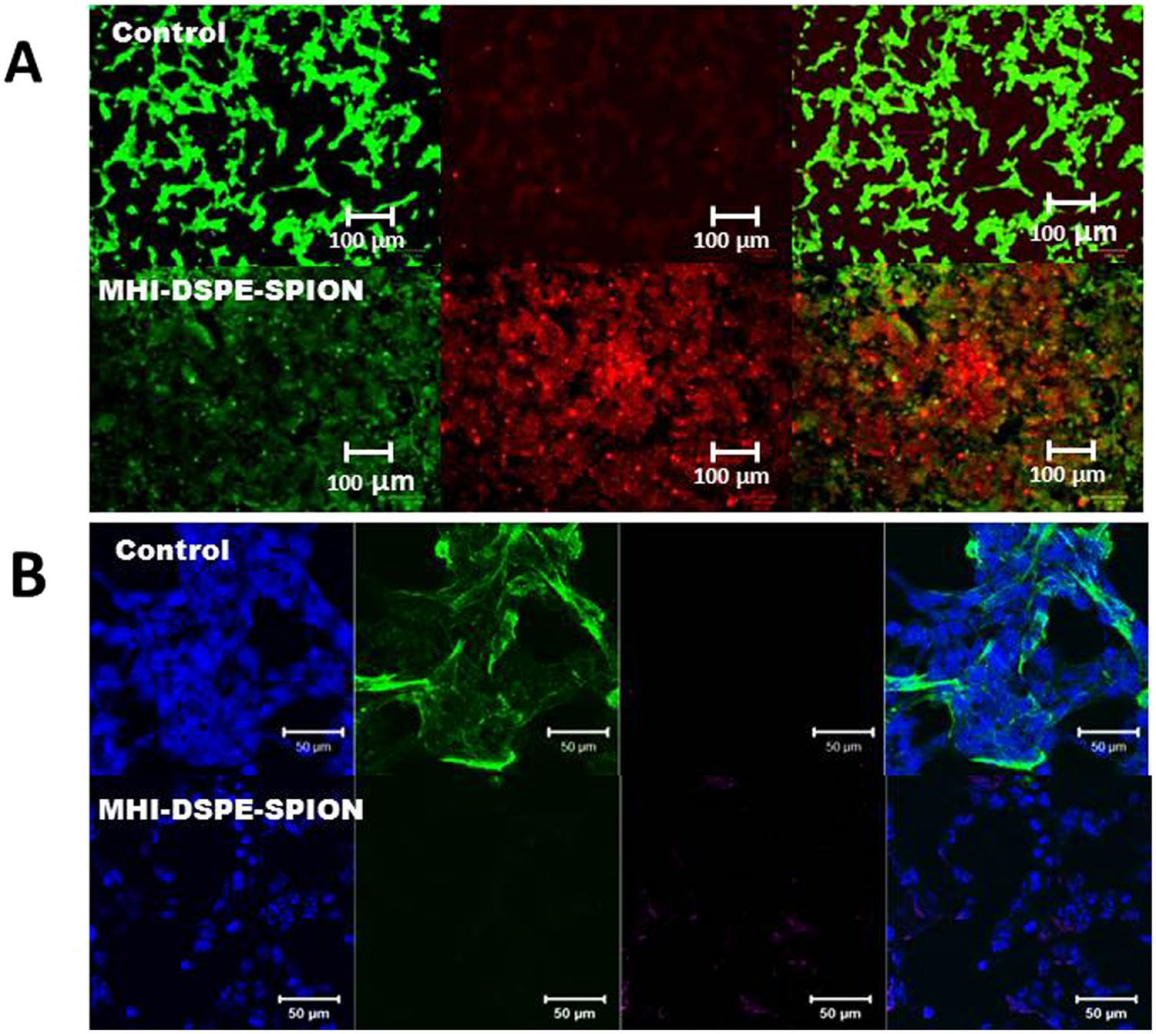
Cell survival of SCC7 cells after incubation and photothermal treatment. (**A**) Qualitative evaluation, by FDA/PI staining, of cell survival upon treatment with MHI-DSPE-SPION plus laser irradiation. Fluorescence images of SCC7 cells after photothermal treatment. Viable cells were stained green with FDA. Dead/later apoptosis cells were stained red with PI. (**B**) Cell actin and nuclei were stained by Alexa 488-labelled secondary antibody binding to the α-sma primary antibody and DAPI, respectively. Control SCC7 cells showing actin expression indicate cell attachment. The MHI-DSPE-SPION-treated SCC7 cells after photothermal treatment show minimal actin expression due to cell detachment.

To confirm the hyperthermia effect on SCC7 cells qualitatively, we conducted a cell cytotoxicity assay using fluorescein diacetate (FDA) and propidium iodide (PI) after 2 hrs of incubation of MHI-DSPE-SPION followed by laser irradiation (1 W/cm^2^) (Fig. 10A). Live cells are represented by green fluorescence after the non-fluorescent compound is converted into the green fluorescent compound “fluorescein”. In contrast, dead cells are shown by red fluorescence due to PI uptake by the cell nucleus, which intercalates with DNA showing red fluorescence. *In vitro* treatment efficiency was directly visualized through staining, and the images illustrate that laser irradiation alone was quite safe for SCC7 cells. To further validate cell viability, the immunocytochemistry of alpha-smooth muscle actin(α-sma) expression was evaluated using anti-α-sma antibody (primary) and donkey anti-rabbit IgG (secondary), which gave green fluorescence from the Alexa flour 488 dye (Fig. 10B). The cell attachment property of SCC7 cells was analyzed with or without MHI-DSPE-SPION under laser irradiation (1 W/cm^2^). After irradiation, cells that were incubated with MHI-DSPE-SPION showed markedly decreased expression of α-sma, indicated by reduced green fluorescence. In contrast, control cells showed a high expression of α-sma. This result confirms that MHI-DSPE-SPION kills cancer cells when irradiated with a laser.

**Figure 11:**
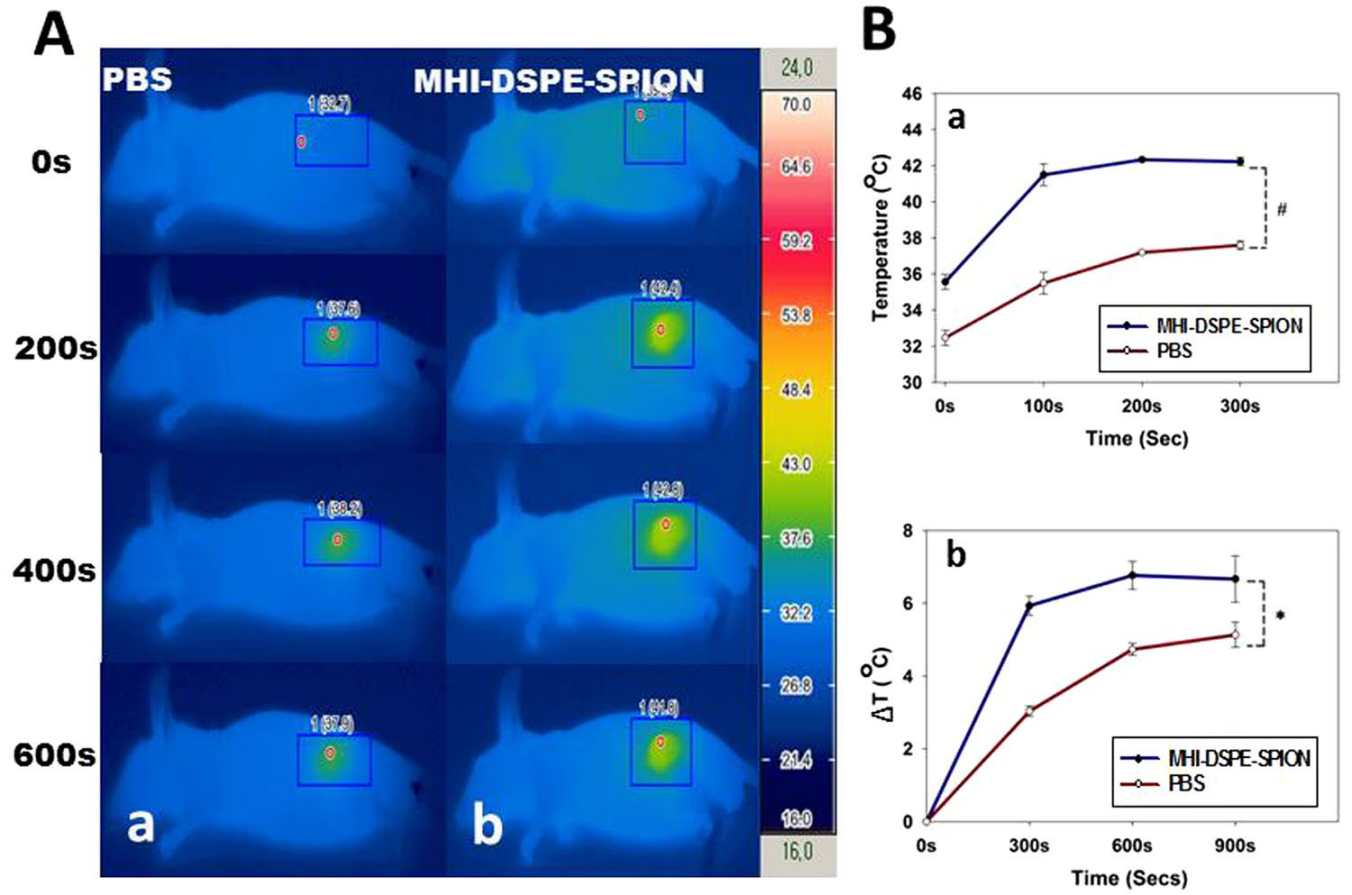
Temperature increase profiles in SCC7 tumor tissue after *in vivo* photothermal treatment. (**A**) Infrared photothermal images of mice measured after tail vein injection and laser irradiation. (a) Mice injected with 200 µL PBS plus further laser irradiation, (b) Mice injected with 200 µL MHI-DSPE-SPION at 10 mg[Fe]/kg concentration in PBS plus further laser irradiation. (**B**) (a) Maximum temperature profiles of SCC7 subcutaneous tumors after tail vein post-injection plus further laser irradiation and (b) Temperature change of tumor area upon laser irradiation. *P < 0.05 relative to PBS injected group (n = 3).

The potential of MHI-DSPE-SPION for PTT was studied using the SCC7 tumor model. On the basis of the tumor accumulation of MHI-DSPE-SPION in observations with fluorescence and MRI, photothermal imaging was performed 3 days post-intravenous injection of MHI-DSPE-SPION. SCC7 tumor were irradiated with an 808 nm laser for 10 min. Upon 100 sec of laser exposure, the average temperature changing of the tumor area reached approximately 6 °C with 1 W/cm^2^ of laser irradiation (Fig. 11A). However, PBS injected mice showed an average temperature change of only 3 °C. At the end of 5 min, laser irradiation of the MHI-DSPE-SPION group achieved an increase of 6 °C in the temperature of the tumor area, whereas the PBS group achieved an increase of 4.4 °C (Fig. 11B). This result demonstrates the ability of MHI-DSPE-SPION to impart the PTT effect, even after a 3-day time period, due to its long-term accumulation. Mitochondria are organelles that are readily susceptible to temperature elevation. MHI-148 is a heptamethine dye whose accumulation in mitochondria is well established^29^. Therefore, the accumulation of MHI-DSPE-SPION in the mitochondria of cells can improve the PTT effect, resulting in better hyperthermia and more enhanced cytotoxicity.

**Figure 12:**
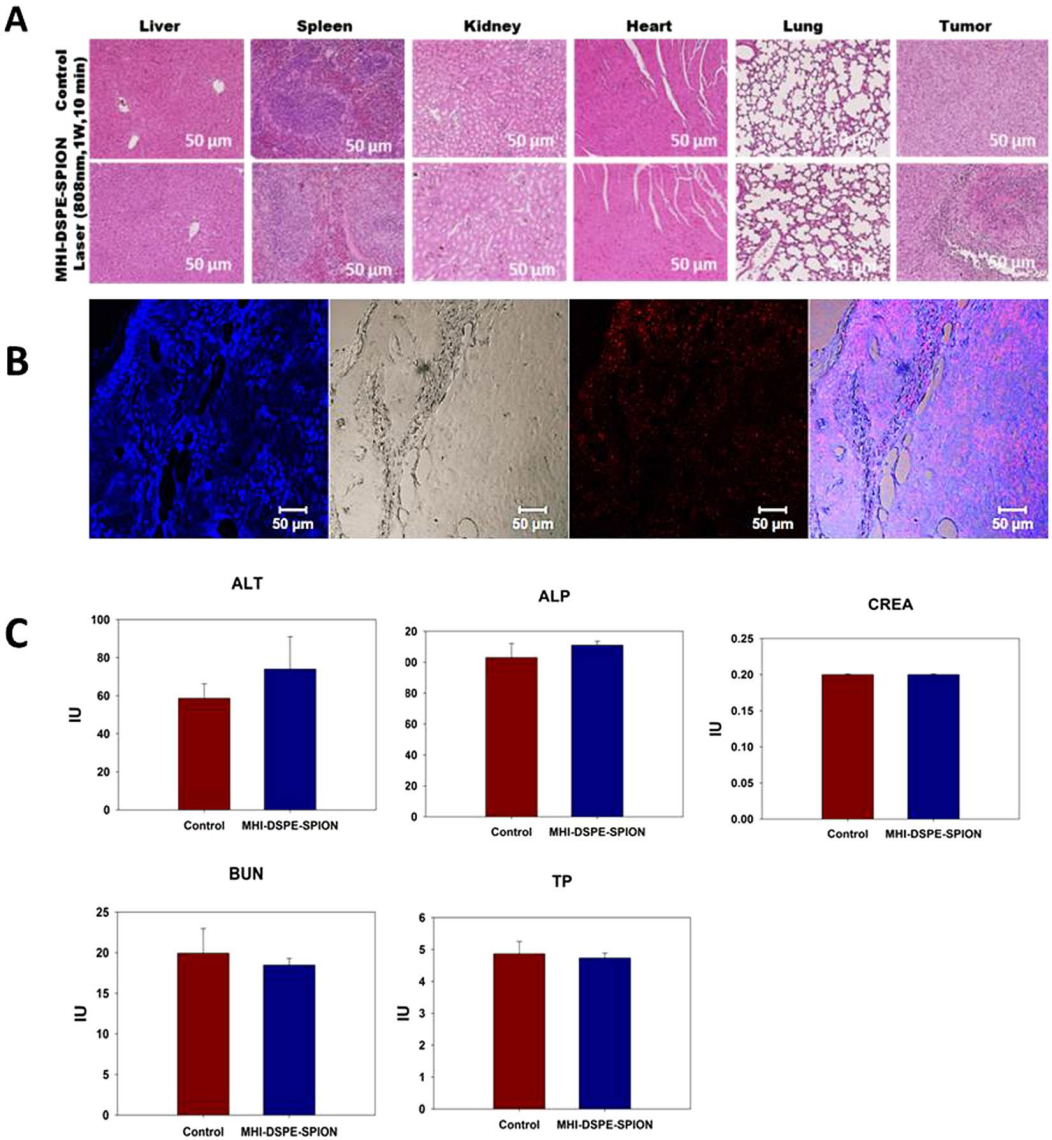
Histological assessment and blood biochemistry analysis of MHI-DSPE-SPION. (**A**) HE stain of five organ tissues (liver, spleen, kidneys, heart and lung) and tumor from the mice treated with MHI-DSPE-SPION, 24 hrs after PTT treatment. (**B**) Confocal fluorescent microscopic analysis of tumor tissue after PTT. Red fluorescence, signifying the presence of MHI-148 in tissue. (**C**) Blood biochemical analysis of alanine aminotransferase (ALT), alkaline phosphatase (ALP), creatinine (CREA), blood urea nitrogen (BUN) and total protein (TP) in BALB/c mice (n = 4). The cardiac puncture method was employed to collect 1 mL of fresh blood from mice.

The intra-tumoral injection of MHI-DSPE-SPION and DSPE-SPION, followed immediately by PTT, were also checked at 1 W/cm^2^ for 10 min. The temperature of the tumor area changed by approximately 20 °C for MHI-DSPE-SPION-injected mice and 5 °C for DSPE-SPION-injected mice at 100 sec (in Figure S4 of the supplementary information). Finally, as illustrated in the H&E-stained organ tissue slices, no significant necrotic cells were observed in various organs for the group treated by the intravenous injection of MHI-DSPE-SPION, which indicates the excellent safety of MHI-DSPE-SPION for *in vivo* application at high dosage. Histological staining of the excised tumors at 24 hrs after injection of MHI-DSPE-SPION under laser irradiation revealed common characteristic features of thermal damage in tumors treated with MHI-DSPE-SPION, such as coagulative necrosis (Fig. 12A). CLSM images of *ex vivo* tumor tissue after 24 hrs of MHI-DSPE-SPION injection showed fluorescence at the NIR/ICG filter, signifying the presence of MHI-148 in tissue (Fig. 12B). For serum protein analysis, blood was taken from mice by cardiac puncture method and analysed for alanine amino transferase (ALT), alkaline phosphatase (ALP), creatinine (CREA), blood urea nitrogen (BUN) and total protein (TP) in BALB/c mice. There was no significant change in MHI-DSPE-SPION group compared to control group where PBS was injected (Fig. 12C).

### Intratumoral injection of MHI-DSPE-SPION for SCC7 tumor reduction

**Figure 13:**
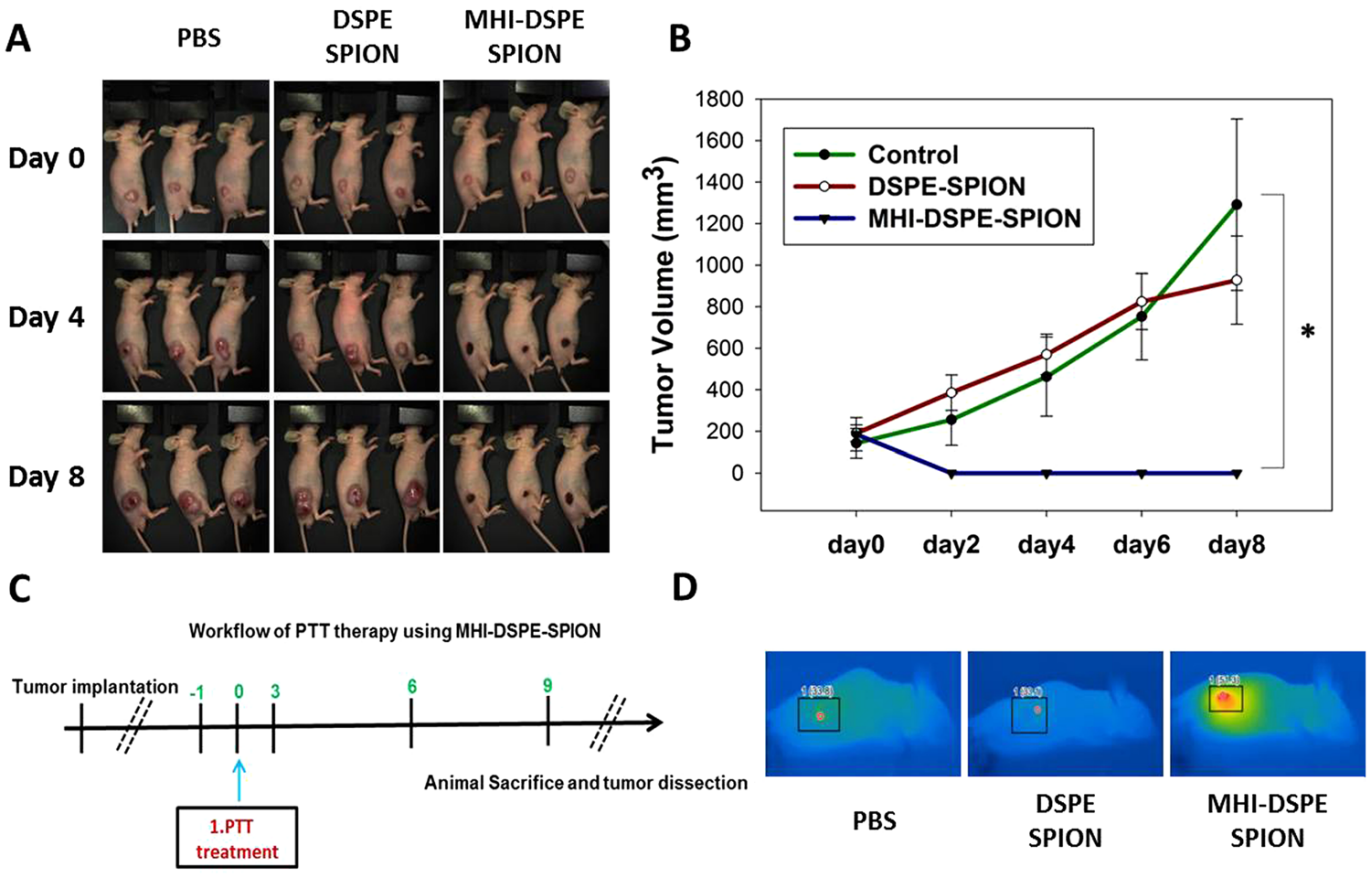
Photothermal mediated tumor reduction study. (**A**) Mice injected with 50 µL PBS, DSPE-SPION (10 mg[Fe]/kg) and MHI-DSPE-SPION (10 mg[Fe]/kg) plus further laser irradiation. (**B**) Tumor growth curves of PBS, DSPE-SPION and MHI-DSPE-SPION. (**C**) Workflow depicting PTT. (**D**) Infrared photothermal images of mice tumor (inset), *P < 0.05 relative to PBS-injected group (n = 3).

After the intratumoral injection of MHI-DSPE-SPION at a concentration of 10 mg[Fe]/kg, the tumor region was irradiated by a 1 W/cm^2^ near-infrared laser for 10 min. PBS and DSPE-SPION were injected as a control, at 10 mg[Fe]/kg and irradiated with 1 W/cm^2^ near-infrared laser for 10 min. Then, the temperature variation of the tumor in 3 different groups was observed by an infrared thermal imaging camera. The maximum temperature of tumors in the MHI-DSPE-SPION, DSPE-SPION, and PBS groups was 50 °C, 33 °C and 33 °C, respectively (Fig. 13). When the tumor was treated with MHI-DSPE-SPION, it reached a temperature that was adequate to destroy and treat the tumor. This demonstrates that accumulation in the tumor was able to increase the temperature of the MHI-DSPE-SPION group.

## Conclusion

A multifunctional nanoplatform was successfully developed using DSPE-PEG-NH_2_, SPION and MHI-148 (MHI-DSPE-SPION). *In vitro* studies revealed that the MHI-DSPE-SPION possesses a good cellular uptake profile in SCC7 and excellent compatibility with the NIH3T3 cell line, even at a concentration of 1 mg/mL. The *in vivo* biodistribution profile of MHI-DSPE-SPION showed a longer accumulation pattern in optical imaging, which can be attributed to active targeting uptake of MHI-DSPE-SPION in the SCC7 tumor.

*In vitro* photothermal analysis showed an excellent PTT effect in the SCC7 cell line, when treated with 100 µg/mL of MHI-DSPE-SPION. *In vivo* application with a photothermal laser increased the temperature of the tumor. MHI-DSPE-SPION possesses a good photothermal heating ability with an inherent MHI dye-mediated targeting property in cancer cells. The advantage of the MHI-DSPE-SPION system, compared to other similar NIRF dye-conjugated/loaded systems, is its active targeting and long-term accumulation property in tumors. A study of the photothermally mediated SCC7 tumor reduction of MHI-DSPE-SPION showed complete tumor ablation and reduction for up to 8 days in the BALB/c nude mice model.

In conclusion, MHI-DSPE-SPION was used as a cancer theranostics material because it provides MRI-optical imaging capabilities and the PTT effect in the SCC7 tumor induced BALB/c nude mice.

## Materials and Methods

### Materials

MHI-148: (2-[2-[2-chloro-3-[2-[1,3-dihydro-3,3-dimethyl-1-(5-carboxypentyl)-2H-indol-2-ylidene]-ethylidene]-1-cyclohexene-1-yl]-ethyl]-3,3-dimethyl-1-(5-carboxypentyl)-3H-indolium bromide was purchased from Bioacts (DKC corporation, Incheon, South Korea). DSPE-PEG2000-Amine from Avanti polar lipids (Alabaster, USA). 1-Ethyl-3-(3-dimethylaminopropyl) carbodiimide (EDC), N-hydroxysuccinimide (NHS) and dicyclohexylcarbodiimide (DCC) were purchased from Sigma Aldrich, St Louis, USA. Dulbecco’s modified Eagle’s medium (DMEM) and RPMI-1640 were purchased from Thermo Scientific, Waltham, USA. 3-(4,5-dimethylthiazol-2-yl)-5-(3-carboxymethoxyphenyl)-2-(4-sulfophenyl)-2H-tetrazolium) (MTS) was purchased from Promega, Madison, USA. All other reagents were of analytical or chromatographic grade.

### Synthesis of DSPE-SPION

DSPE-PEG-SPION was made by the solvent hydration method^15^. Briefly, 10 mg of DSPE-PEG_2000_-Amine was dissolved in 800 μL chloroform. Oleic-acid-coated SPION was dissolved in 200 μL chloroform and added to DSPE-PEG_2000_ at ratios of 10:2 and 10:4 (DSPE-PEG_2000_ : SPION weight ratio). Chloroform was evaporated completed in dry hood at room temperature. 3 mL 10 mM HEPES buffer saline was added to the thin film of DSPE-PEG_2000_ and SPION and was mixed and probe-sonicated with 22% amplitude and a 2:3 pulse ratio for 2 mins, keeping the solution at 4 °C. A Vivaspin 20 ultrafiltration column of size 100 kDa (Sartorius, Goettingen, Germany) was used to remove unloaded SPION from the SPION-DSPE-PEG_2000_.

### Synthesis of MHI-DSPE-SPION

Conjugation of DSPE-SPION to MHI-148 was achieved by simple EDC-NHS chemistry. Briefly, DSPE-SPION (2.5 mg) was suspended in 1 mL distilled water and then 50 μg of MHI-148 in 25 μL DMSO was added to it, followed by 1-Ethyl-3-(3-dimethylaminopropyl) carbodiimide (EDC) and N-Hydroxysulfosuccinimide sodium salt (Sulfo-NHS) in ten molar excess compared to MHI-148. The solution was mixed at 4 °C for 2 days and dialyzed in a 3500 Da membrane (Spectra/Por, California, USA) for 3 days to remove excess DMSO, unconjugated MHI-148, and EDC/NHS.

### Characterization of MHI-DSPE-SPION

The morphology of MHI-DSPE-SPION was visualized by transmission electron microscopy (TEM). The hydrodynamic size of DSPE-SPION and MHI-DSPE-SPION was measured using DLS, and nanoparticle charge was measured on a Zetasizer instrument (Nano-Z590, Malvern Instruments, Worcestershire, UK). FTIR analysis was conducted to study the conjugation of MHI to DSPE-SPION and coating of SPION by DSPE-PEG.

### Analysis of SPION concentration in MHI-DSPE-SPION

The ferrozine assay method was used to analyze the iron concentration in DSPE-SPION. Briefly, DSPE-SPION (50 μL) was added to 12 M HCL (50 μL) and incubated for 40 min. Then, 50 μL of 4 M ammonium acetate, 240 μL of 2 M NaOH and 5% hydroxylamine HCl were added sequentially and incubated for another 30 min. Finally, 0.02% Ferrozine® solution was added to obtain a purple-colored complex, whose absorbance was read at 560 nm using a multiplat reader (Tecan, Austria GmbH, Grödig, Austria).

### Absorbance and emission spectra of MHI-DSPE-SPION

The absorbance spectra of MHI-148 dye and MHI-DSPE-SPION were acquired on a UV/Visible spectrophotometer (Shimadzu Corp., Kyoto, Japan). Fluorescence emission was analyzed using a fluorimeter (Scinco, Seoul, Korea) keeping ABS_max_ at 774 nm.

### *In vitro* cytocompatibility study

The cytocompatibility of MHI-DSPE-SPION against NIH3T3 cells was measured by MTS assay. 10^4^ cells/well were cultured in a CO_2_ incubator in 96-well plate at 37 °C in a humidified environment for one day. MHI-DSPE-SPION was added to the cells in quadruplicate to analyze the cytocompatibility over the concentration range from 0.001 μg/mL to 1000 μg/mL. The cells were incubated for 24 hrs after treatment. Then MTS reagent was added to each of the treated wells with 4 hrs of incubation. Finally, the absorbance was measured at 490 nm, using a microplate reader.

### Cell uptake studies of MHI-DSPE-SPION

SCC7 cells were seeded at a cell density of (5 × 10^4^ cells/well) in an 8-well chamber slide (Lab-Tek2, Hatfield, USA) and supplemented with the respective culture medium for 1 day in a humidified environment and CO_2_ at 37 °C. On the next day, MHI-DSPE-SPION was added to the cells at a concentration of 50 μg/mL for SCC7 cells. After an incubation period of 2 hrs, the cells were washed three times with warm PBS and visualized in a laser confocal microscope (Zeiss LSM 510, Oberkochen, Germany) in a Cy5.5 filter.

The Prussian blue technique was utilized to study the cell uptake profile of MHI-DSPE-SPION. In this technique, iron content imparts a blue color and indicates any attachment and uptake of MHI-DSPE-SPION in SCC7 cells. 5 × 10^4^ cells/well were seeded in an 8-well chamber slide (Lab-Tek2, Utah, USA) with RPMI medium (Thermo Scientific, Utah, USA), in a humidified 5% CO_2_ atmosphere, overnight at 37 °C. On the next day, the media were removed and two PBS washes were applied; 50 μg/mL of MHI-DSPE-SPION was added to SCC7 cells and incubated for 1 hr. The cells were fixed with 500 μL of PFA (4%) for 10 min and washed with PBS. To each well, 100 μL of 4% potassium ferrocyanide (II) trihydrate and 4% HCl solution (in PBS) was added, followed by incubation for 20 min. After washing thoroughly with PBS, cells were counter-stained with nuclear fast red (50 μL/well) for 5 min. SCC7 cells with blue stained profile represented MHI-DSPE-SPION uptake. Images were collected on an inverted light microscope.

### T2 relaxivity of MHI-DSPE-SPION

MHI-DSPE-SPION was analyzed over 4 dilutions in a 1.5 mL centrifugation tube, using a 2-fold serial dilution, starting from a concentration of 0.25 mM [Fe]. MRI experiments were employed to obtain the T2 relaxation time and T2 relaxivity coefficient, using a 3.0 T clinical MRI scanner (MAGNETOM Tim Trio, Siemens Medical Solutions, Erlangen, Germany) with TR (repetition time) and TE (echo time), as follows: TR = 1950 ms; TE = 14, 28, 41, 55, 69, 83, 97, 110, 124, 138, 152, 167, 179, 193, 207, 220 ms.

### *In vitro* MRI of MHI-DSPE-SPION

MRI of the phantom tube was conducted by a T2-weighted imaging protocol based on SPION concentration. SCC7 cells were seeded at a density of 1 × 10^6^ cells/well in a 6-well plate, and incubated in a humidified environment in CO_2_ at 37 °C for one day. RPMI medium containing 10 vol% FBS and 1 wt% of antibiotic was added to culture the cells. MHI-DSPE-SPION was added at 50 μg[Fe]/mL and 100 μg[Fe]/mL concentrations, and incubated for 2 hrs. The cells were washed with PBS and fixed with 4% formaldehyde for 10 min and then harvested into 1.5 mL tubes, in which 10% gelatin was used as tissue mimic. The tubes were placed perpendicular to the main magnetic induction field (B0) in a water bath. MRI was performed on a 3 T clinical MRI scanner (Magnetom Tim Trio, Siemens Medical Solutions, Erlangen, Germany). The MRI measurement parameters were as follows. Time of repetition: 3000 ms, time of echo: 83 ms, flip angle, 150°, echo train length: 12, field of view: 150 mm × 150 mm, section thickness: 2 mm, matrix number: 448 × 314.

### Animal tumor model

Male BALB/c nude mice (6 weeks old and weighted 18–20 g) were purchased from Jungang Lab Animal, Inc., Korea. All experiments were performed in accordance with relevant guidelines and regulations. The Chonnam National University Medical School Research Institutional Animal Care and Use Committee approved the experimental protocol (CNUHH 2014–148). To obtain the tumor model, 2 × 10^6^ SCC7 cells were injected into the mice through subcutaneous injection. The tumor volume was calculated as L × B^2^/2, where L and B correspond to the major and minor axes of the tumor.

### *In vivo* biodistribution of MHI-DSPE-SPION

When tumors reached approximately 5–7 mm in size (after approximately 15 days), MHI-148-conjugated DSPE-PEG-SPION (in PBS, 200 μL) at a concentration of 10 mg [Fe]/kg was intravenously injected into each mouse through the tail vein such that the MHI-148 dosage in mice was 0.2 mg/kg. The whole body was imaged at 2 hrs, 4 hrs, 1 day, 2 days, 3 days, and 6 days on the aforementioned tumor-bearing nude mice. The tumor was imaged simultaneously with MRI and FOBI in an *in vivo* imaging system (Neoscience, Korea) with 825-nm emission filters at an exposure time of 1 to analyze the *in vivo* bio-distribution of MHI-148-conjugated DSPE-PEG-SPION.

### *In vivo* MRI of MHI-DSPE-SPION and DSPE-SPION

Athymic mice (nu/nu-ncr, BALB/c mice) (5–6 weeks old, 20–25 g) were purchased from Jungang Lab Animal (Inc., Korea). Experimental protocol was approved by Chonnam National University Medical School Research Institutional Animal Care and Use Committee (CNUHH 2014-148). The mice were injected with 2 × 10^6^ SCC7 cancer cells subcutaneously. After 2 weeks, the SCC7 tumor was formed (7–9 mm^3^ in size). MRI was conducted on a 3.0 T clinical MRI scanner (MAGNETOM Tim Trio, Siemens Medical Solutions, Erlangen, Germany) using a volume coil (Rapid Biomedical GmbH). The MRI signal intensities were measured using the Maro view image viewer (Marotech Inc., Korea). Mice were injected with MHI-148-conjugated DSPE-PEG-SPION or DSPE-PEG-SPION (10.0 mg[Fe]/kg) via tail vein. T2-weighted MRI was performed as follows: TR/TE, 3000/61; flip angle, 145°; field of view, 41 × 41 mm; slice thickness, 1.0 mm; gap, 20% of slice thickness; and matrix, 256 × 205.

### *In vitro* hyperthermia

MHI-DSPE-SPION (containing 100 µg/mL and 40 µg/mL and 0 µg/mL of MHI-148) was added into different 1.5mL centrifuge tubes (Eppendorf, Hamburg, Germany) in triplicate, in PBS. MHI-DSPE-SPION was irradiated using a 1 W/cm^2^ laser power for 300 sec. The temperature changes of each group were recorded by an infrared thermal imaging camera (Avio IR camera/Thermometer, Shinagawa-ku, Tokyo). To measure the photothermal efficiency, MHI-DSPE-SPION was diluted into 20 µg/mL [MHI-148] concentrations with PBS and exposed to an 808 nm NIR laser for 5 min with different laser powers (0.4 W/cm^2^, 0.6 W/cm^2^, 0.8 W/cm^2^, 1 W/cm^2^). Finally, the photothermal stability of MHI-DSPE-SPION was observed in six cycles of laser irradiation at 1 W/cm^2^ for 40 µg/mL [MHI-148]. Temperature elevations of 15 °C, 14 °C, and 13 °C were observed for MHI-DSPE-SPION.

### FDA/PI and α-sma expression after PTT

SCC7 cells were seeded in 8-well chamber slides (Lab-Tek2, USA) and cultured in RPMI medium in a 5% CO_2_ incubator at 37 °C in a humidified environment. After one day of incubation, 100 μg/mL of MHI-DSPE-SPION was added, followed by 2 hrs of incubation. The wells were washed with warm PBS 3 times and subjected to a PTT laser (808 nm, 1 W) for 5 min. After hyperthermia, MHI-DSPE-SPION was treated with 8 μL of 4 μM *fluorescein diacetate* (FDA) and 0.5 μM propidium iodide (PI) dissolved in PBS for 5 min at room temperature. The coverslip was directly viewed under a fluorescent microscope, using a standard fluorescein bandpass filter for FDA and a Texas Red® dye filter for PI.

The manufacturer’s protocol was followed for immunocytochemistry (Abcam, UK) expression analysis of alpha-smooth muscle actin (α-sma). Primary and secondary antibodies were was diluted to 1/250 (0.8 μg/mL) and 1/500 (4 μg/mL), respectively. The sample group, after being exposed to a PTT laser (808 nm, 20 W, for 5 min), was washed with warm PBS and fixed using ice-cold methanol for 10 min. After fixation, the cells were washed 3 times in PBS and incubated in 0.02% Triton X-100 for 10 min to increase cell permeability. Next, the cells were incubated in 1% BSA in PBST for 1 hr to avoid any non-specific binding of the primary antibody. The cells were incubated in 100 μL primary antibody solution overnight at 4 °C, and washed again in PBS. Then, the secondary antibody was added and incubated for 1 hr in the dark. Finally, the cells were washed again in PBS, dried at room temperature, and mounted using Prolong DAPI gold. The cells were observed by a confocal laser scanning microscope (CLSM).

### *In vivo* photothermal therapy of tumor

After tumor modeling was performed and the tumor sizes reached 200 mm^3^, the mice were divided into three groups (three per group). The mice were separately injected with 50 μL PBS, 50 μL DSPE-SPION (10 mg[Fe]/kg), and 50 μL MHI-DSPE-SPION (10 mg[Fe]/kg). All groups were irradiated by a 808 nm laser (1 W/cm^2^) for 10 min, 24 hrs after injection. Changes of tumor size for every mouse were recorded within 8 days. Mice with tumor volumes exceeding 1500 cm^3^ were euthanatized according to animal protocol.

### Histopathology and blood biochemical analysis

SCC7 tumor-bearing BALB/c mice were injected with 10 mg [Fe]/kg of MHI-148-conjugated DSPE-PEG-SPION. After one day, the tumor was excised and stored in 4% paraformaldehyde for 24 hrs. Paraffin blocks of the tumor were made and sectioned at 5μm, for Prussian blue and fluorescence microscopic analyses. Prussian blue staining was conducted to analyze the amount of SPION accumulated on day 1 in the tumor and liver. The initial sections were deparaffinized by 100% xylene treatment for one hr, hydrated again by serially immersing in 100%, 95%, 90%, 80%, and 70% ethanol, and finally, washed with flowing tap water for 5 mins. Next, Prussian blue staining was conducted by adding 200 μL of 4% potassium ferrocyanide (II) trihydrate and 4% HCl solution (in PBS), followed by incubation for 40 mins. The sections were counter-stained with nuclear fast red solution for 5 mins to visualize the nuclei.

For H&E staining, BALB/c nude mice were intravenously injected with 10 mg[Fe]/kg of MHI-DSPE-SPION. One group was irradiated with the 808 nm laser (1 W/ cm^2^) for 10 min, while the PBS-only group was set as the control and killed at 24 hrs after injection. The major organs were harvested and fixed in 10% neutral buffered formalin. Organs were embedded in paraffin and sectioned at 8 μm thickness. H&E were done with slices of organs and tissues, including the liver, spleen, kidney, heart, and lung, and of the tumor were examined using an inverted fluorescence microscope (Olympus iX71, Tokyo, Japan) equipped with an Olympus DP72 camera.

For the *ex vivo* histofluorescence assay, MHI-148 dye was observed by mounting deparaffinized and hydrated sections by GOLD/DAPI mounting medium and viewed in a confocal microscope (Zeiss LSM 510, Oberkochen, Germany). For blood biochemical analysis, BALB/c mice were injected intravenously with 100 μL of 10 mg[Fe]/kg of MHI-DSPE-SPION (n = 4) or PBS (n = 4) as a control. Mice were euthanised at day 7 to collect blood (1 mL) for biochemical analysis of liver function markers, including alkaline phosphatase (ALP), alanine aminotransferase (ALT), as well as the kidney function markers creatinine (CREA) and blood urea nitrogen (BUN) and total protein (TP).

## Electronic supplementary material

Supplementary
