# Supplementary material for: Near-Infrared Heptamethine Cyanine Based Iron Oxide Nanoparticles for Tumor Targeted Multimodal Imaging and Photothermal Therapy
Source: Sci Rep. 2017 May 18;7:2108. doi: 10.1038/s41598-017-01108-5 (PMC5437012; doi:10.1038/s41598-017-01108-5)
Supplement: Supplementary file 1 — Supplementary [file 41598_2017_1108_MOESM1_ESM.pdf]

Near-Infrared Heptamethine Cyanine Based Iron Oxide Nanoparticles for Tumor Targeted Multimodal Imaging and Photothermal Therapy

*Sejy Lee[1#], Reju George Thomas[1#], Myeong Ju Moon[1], Hyeong Ju Park[2], In-Kyu Park[3], Byeong-Il Lee[2], Yong Yeon Jeong[1]**

[1]Department of Radiology, Chonnam National University Hwasun Hospital, Hwasun 58128, South Korea,

[2] Medical Photonics Research Center, Korea Photonics Technology Institute, Gwangju 61007, South Korea

[3]Department of Biomedical Sciences,Chonnam National University Medical School, Gwangju 61469, South Korea

E-Mail: yjeong@jnu.ac.kr

[#] The two authors equally contributed to this work

*Author to whom correspondence should be addressed; E-Mail: yjeong@jnu.ac.kr;

Tel.: +82-61-379-7102; Fax: +82-91-379-7133.

Supplementary

In vivo MR imaging of DSPE-SPION

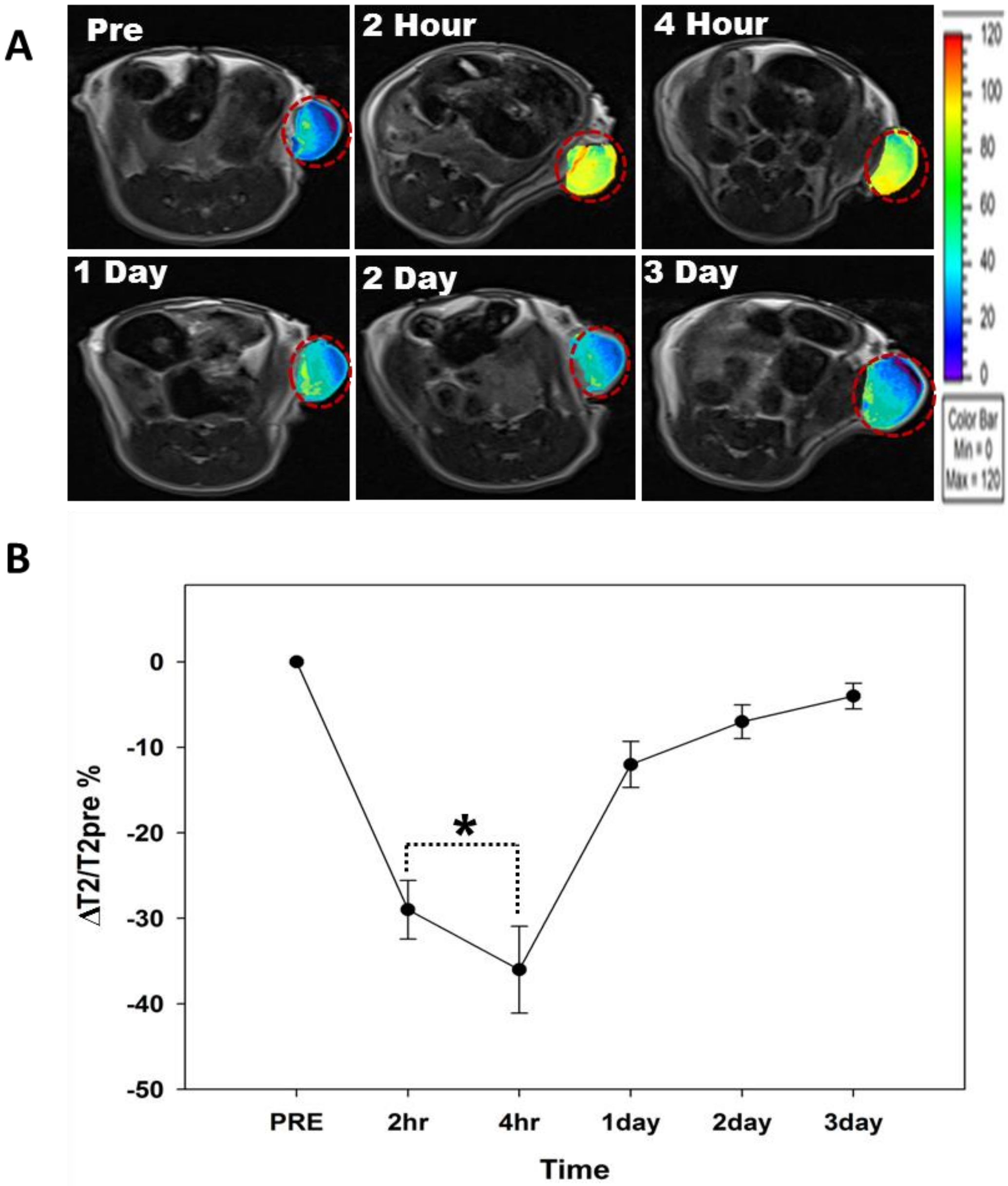

Figure S1. In vivo MR imaging of SCC7 tumor. (A) T2-weighted MR imaging of DSPE-SPION. (B) The signal intensity change of T2-weighted MR imaging measured at different time point (pre, 2hr, 4hr, 1day, 2day, 3day) by 10 mg/kg[Fe]DSPE-SPION (in PBS) injected through tail vein. *P <0.05 relative to the pre injection T2 contrast (n=3).

***Ex vivo* Prussian blue staining**

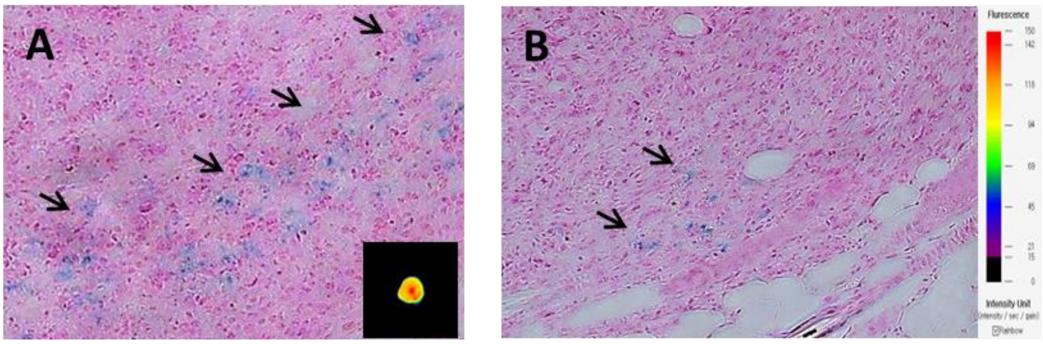

Figure S2. Prussian blue staining in *ex vivo* SCC7 tumor at 1 day. (A) MHI-DSPE-SPION intravenously injected at 10 mg/kg [Fe] concentration in 200 μl PBS. (B) DSPE-SPION intravenously injected at 10 mg/kg [Fe] concentration in 200 μl PBS. The uptake of SPION in SCC7 tumor cell shows blue color in Prussian blue staining (arrows).

Photothermal studies of DSPE-SPION

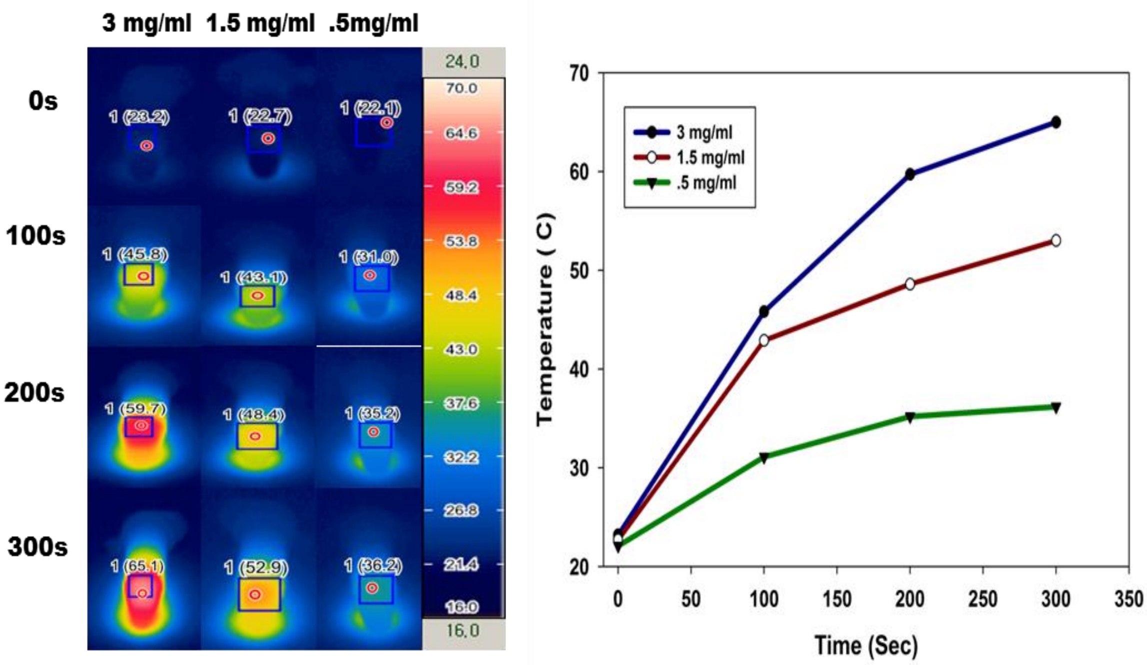

Figure S3. Photothermal studies of DSPE-SPION (a) Temperature rise after laser irradiation of different concentrations of DSPE-SPION. (b) Quantification of temperature rise after laser irradiation of different concentrations of DSPE-SPION. 2W/cm2 laser was used for this experiment.

***In vivo* PTT intra-tumoral injection of MHI-DSPE-SPION and DSPE-SPION**

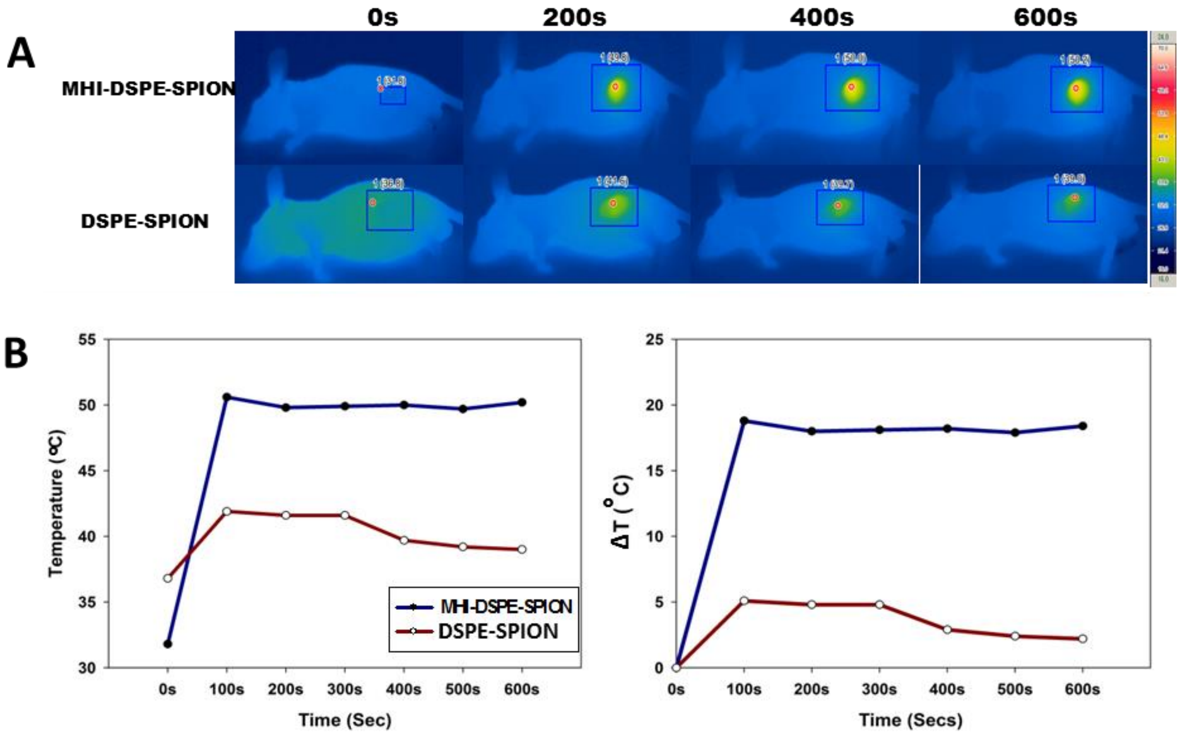

Figure S4. Temperature increasing profiles in SCC7 tumour tissue after *in vivo* photothermal treatment. (A) Infrared photothermal images of partial mice measured after intra tumor injection and laser irradiation. The intratumoral injection with 100 μL MHI-DSPE-SPION at 10mg/kg [Fe] concentration in PBS and 100 μL DSPE-SPION at 10mg/kg [Fe] plus further laser irradiation. (B) Maximum temperature profiles of SCC7 subcutaneous tumors after tail vein post injection plus further laser irradiation. (C) Temperature change of tumor area upon laser irradiation.
